# Effectiveness of recommendations in promoting the use of mobile health applications in health guidance: a randomized controlled trial

**DOI:** 10.1093/joccuh/uiaf036

**Published:** 2025-07-17

**Authors:** Takeshi Onoue, Kazuki Nishida, Yoshio Nakata, Fumi Hayashi, Miki Marutani, Naoki Sakane, Jiro Moriguchi, Shigeki Muto, Kiminori Kato, Izuru Masuda, Tomonori Okamura, Keiichi Matsuzaki, Takashi Kawamura, Kazuyo Tsushita

**Affiliations:** Department of Endocrinology and Diabetes, Nagoya University Graduate School of Medicine, Nagoya, Japan; Department of Advanced Medicine, Nagoya University Hospital, Nagoya, Japan; Institute of Health and Sport Sciences, University of Tsukuba, Tsukuba, Japan; Graduate School of Nutrition Sciences, Kagawa Nutrition University, Saitama, Japan; Department of Health Promotion, National Institute of Public Health, Saitama, Japan; Division of Preventive Medicine, Clinical Research Institute for Endocrine and Metabolic Disease, National Hospital Organization, Kyoto Medical Center, Kyoto, Japan; Occupational Health Research Center, Kyoto Industrial Health Association, Kyoto, Japan; Department of Health Care, Seirei Center for Health Promotion and Preventive Medicine, Hamamatsu, Japan; Department of Prevention of Noncommunicable Diseases and Promotion of Health Checkup, Niigata University Graduate School of Medical and Dental Sciences, Niigata, Japan; Ningen Dock Center, Mitsubishi Kyoto Hospital, Kyoto, Japan; Department of Preventive Medicine and Public Health, Keio University School of Medicine, Tokyo, Japan; Department of Public Health, Kitasato University School of Medicine, Kanagawa, Japan; Department of Preventive Services, Kyoto University School of Public Health, Kyoto, Japan; National Hospital Organization, Kyoto Medical Center, Kyoto, Japan; Graduate School of Nutrition Sciences, Kagawa Nutrition University, Saitama, Japan

**Keywords:** smartphone application, health guidance, metabolic syndrome, obesity, mobile health application

## Abstract

**Objectives**: Use of commercially available mobile health (mHealth) applications in supporting lifestyle improvements has become popular in recent years. However, the effectiveness of advice promoting the use of such applications based on individual behavioral goals in a health guidance setting remains unclear. This study explored how guiding participants of the Specific Health Guidance (SHG) program, a Japanese public health initiative to prevent cardiovascular disease, to use commercially available mHealth applications impacted their application usage, lifestyle habits, and cardiovascular risk factors.

**Methods**: In this multicenter, randomized, open-label, parallel-group comparison study, 156 individuals with a history of SHG participation and who were engaged in the Motivational Health Guidance program (a type of SHG) in 2021 were assigned to intervention (*n* = 76) or control (*n* = 80) groups. Whereas both groups received standard guidance, the intervention group also received recommendations for mHealth applications based on their individual behavioral goals. The participants’ application usage, behavioral changes, and body weight were assessed after 3 months, with health checkup data evaluated after 1 year.

**Results**: The proportion of mHealth application users after 3 months was significantly higher in the intervention group (68.4%) than in the control group (40.0%). The intervention group also reported a significantly greater weekly frequency of mHealth application usage. Moreover, the intervention group reported a significantly decreased change in triglyceride levels after 1 year compared with the control group.

**Conclusions**: Recommending commercially available mHealth applications in a health guidance setting significantly increased the number of mHealth application users and their frequency of use.

## Teaser

Key points

- Commercially available mobile health (mHealth) applications for lifestyle improvements have gained in popularity.
- However, in health guidance settings such as occupational health programs, the effectiveness of recommending available mHealth applications based on individual behavioral goals remains unclear.
- This study demonstrated that providing advice regarding mHealth applications for individual behavioral goals in a health guidance setting significantly increased the number of users of mHealth applications and their frequency of use, and improved participants’ triglyceride levels.
- The use of commercially available mHealth applications may enhance the effectiveness of health guidance programs, especially in environments with limited human and financial resources.

## 1. Introduction

The prevention of cardiovascular disease is crucial for extending people’s healthy lifespan and optimizing health care costs. In Japan, the Specific Health Guidance (SHG) program, a national system, was established in 2008 to identify individuals with metabolic syndrome in health checkups and provide appropriate health guidance to them, mainly targeting the working-age population.^1^ SHG is a key initiative for companies promoting corporate health management and is occasionally implemented as part of occupational health activities in workplace settings. This program includes the following service types: (1) Intensive Health Guidance, which provides over 3 months of continuous support, and (2) Motivational Health Guidance, which only provides initial support with subsequent self-management. These differing approaches are provided based on the specific severity of metabolic syndrome. According to previous studies, individuals who participate in the SHG program report considerable reduction in weight and waist circumference as well as a suppression of glycated hemoglobin (HbA1c) elevation compared with those who do not participate in the program.^2^ Specifically, the degree of weight loss following participation in the SHG program has been associated with improvements in risk factors for atherosclerotic cardiovascular disease.^3^

However, some individuals who smoke, do not exercise, or tend to eat late at night do not report benefits from conventional SHG.^4^^,^^5^ Additionally, an analysis of our SHG databases indicates that the effectiveness of the Motivational Health Guidance program diminishes for those who had previously participated in the SHG program.^6^ Second-round advice may not effectively motivate these participants. In environments with limited human and financial resources, such as Motivational Health Guidance, combining other interventions may be more effective than increasing the number of guidance sessions.

Recently, numerous smartphone applications supporting lifestyle improvements have been developed and disseminated commercially. The development of some of these mobile health (mHealth) applications has been supervised by health care professionals, ensuring their quality.^7^^,^^8^ Due to the intense market competition, continuous improvements are made to their user interfaces to make them more user-friendly. The use of such commercially available mHealth applications based on individuals’ behavioral goals can be useful in improving and maintaining the lifestyles of those receiving health guidance. Although there have been several studies on the support effects of individual applications,^9-12^ few studies have investigated the effectiveness of introducing and recommending multiple existing applications based on individual characteristics, complications, lifestyle habits, and behavioral goals. Given the widespread adoption of commercially available mHealth applications, introducing and recommending a variety of applications based on individual behavioral goals represents a practical option for health care professionals rather than relying on a single application.

This study investigated the impact of recommending the use of commercially available mHealth applications based on participants’ individual behavioral goals in Motivational Health Guidance, targeting those with a history of participating in SHG programs who are expected to be less responsive to standard health guidance.

## 2. Methods

### 2.1. Trial design

This multicenter, randomized, open-label, parallel-group comparison study, which was called the Download Useful Kind of Application for Your Health Study, was conducted at 5 health guidance institutions in the Kanto, Chubu, and Kansai regions of Japan. Of the 5 participating health guidance institutions, 2 were hospital-affiliated health checkup centers, 2 were companies specializing in health checkups, and 1 was an employee benefits company providing SHG services.

### 2.2. Ethical considerations

The study protocol was approved by the Ethics Committee of Kagawa Nutrition University (No. 315). The clinical trial was registered in the Japanese University Hospital Medical Information Network Clinical Trials Registry (UMIN-CTR: UMIN000042986; URL: https://center6.umin.ac.jp/cgi-open-bin/ctr_e/ctr_view.cgi?recptno=R000049078). Written informed consent was obtained from all participants after providing a detailed explanation of the study’s purpose and methods as well as potential risks and benefits. The study adhered to the ethical principles stipulated in the Declaration of Helsinki and Ethical Guidelines for Medical and Health Research Involving Human Subjects in Japan. This study was not designed to prove the superiority of any specific application, and no conflicts of interest were reported among the developers, interventionists, or evaluators.

### 2.3. Participants

The inclusion criteria were health checkup attendees who (1) were aged between 40 and 64 years; (2) were participants in the Motivational Health Guidance program at 1 of the 5 health guidance institutions between May 21 and December 31, 2021; (3) had a history of participating in SHG programs; (4) were not taking any medication for diseases considered to be cardiovascular risk factors, such as diabetes, hypertension, or dyslipidemia; and (5) regularly used a smartphone. Individuals were excluded if they (1) had a history of heart disease, severe liver or kidney disorders, or malignant tumors within the previous 5 years; (2) had a concurrent infection; (3) had an implanted medical device such as a pacemaker; or (4) were judged by their physicians to be unsuitable for this study due to another medical condition.

### 2.4. Randomization and masking

The participants were randomly assigned to the intervention or control group using sealed, opaque envelopes. The allocation manager pregenerated the sequence using a random number table, and envelopes were distributed to the institutions based on the expected enrollment. Variable block randomization was used with stratification by institution only. Other stratification factors, such as age and sex, were not used due to the small number of participants expected in some strata. Each participant received an envelope in numerical order, with assignments recorded before they were opened. Once given, envelopes could not be retracted. The institutions reported registrations within 14 days. Due to the nature of the intervention, blinding was not feasible, and the participants, investigators, and study staff were not masked to the group allocation.

### 2.5. Intervention

Both groups participated in the standard Motivational Health Guidance program, as detailed in previous reports.^2^^,^^13^ As part of the program, the participants set individual behavioral goals. In addition to the standard program, the intervention group received advice concerning the use of mHealth applications based on their determined behavioral goals during the single health guidance session at the start of the program. The applications used in the intervention included the following 6 types: exercise (promoting various physical activities), walking, diet, reduction of alcohol consumption, peer support, and body measurement (Table 1). These applications were adopted based on their availability, free or low-cost nature, prior use in real-world health guidance settings, balanced coverage across key health areas, scientifically documented or evaluated efficacy,^14-16^ and the developers’ willingness to cooperate with the study. The details of the applications are provided in Supplementary Material 1. The applications were recommended using the Study Guide algorithm (Supplementary Material 2),^17^ which tailored the selection process to the participants’ chosen behavioral goals while considering their individual lifestyle issues, level of motivation, and digital literacy. For example, for participants who sought to improve their diet, “CALO mama Plus” was recommended to those who wanted to completely engage in the program, “Minchalle” to those who wanted peer support but found detailed inputs cumbersome, and “Bodygram” for those who preferred to periodically evaluate their body measurements rather than engaging in continuous diet monitoring. Ultimately, the participants themselves made the final decision regarding whether to use an application and, if so, which one. In addition to being introduced to the mHealth applications, the participants were provided with guidance on downloading and setting up the applications on their smartphones. The specific procedures and support strategies were standardized and described in detail in the Study Guide Book.^17^ Although some applications included paid features, research-specific accounts were provided during the intervention period, and the associated costs were covered by the study budget. Participants in the control group received only standard health guidance. Although the use or recommendation of mHealth applications was not explicitly prohibited, no introduction, recommendation, or support related to the study mHealth applications was provided as part of the intervention protocol.

**Table 1:** mHealth applications used in the study.

**Relevant behavioral goals**	**Application**	**Developer**
Exercise Various physical activities	BeatFit	https://www.beatfit.jp/ Aristol Inc, Tokyo, Japan
Exercise Walking	Aruku&	https://www.arukuto.jp/ ONE COMPATH CO., LTD, Tokyo, Japan
Diet	CALO mama Plus	https://calomama.com/ Wellmira Inc, Tokyo, Japan
Alcohol consumption reduction	Genshu Nikki	https://gen-shu.jp/app/ Otsuka Pharmaceutical Co., Ltd, Tokyo, Japan
Peer support	Minchalle	https://minchalle.com/ A10 Lab Inc, Tokyo, Japan
Body weight	Bodygram	https://bodygram.com/ Bodygram Inc, Tokyo, Japan

Following typical procedures in Motivational Health Guidance, the participants received health guidance from health care professionals in a single health guidance session at the start of the program, with the effectiveness of this guidance evaluated 3 months later. A questionnaire survey regarding the usage of mHealth applications was conducted before the intervention and 3 months later. The participants’ body weights and lifestyle habits were also assessed 3 months later using a questionnaire survey. Data on anthropometric measurements (including body weight) and laboratory test results at baseline and 1 year were collected from the Specific Health Checkups that were conducted in the baseline year and the following year.

### 2.6. Outcomes

The primary outcome was the usage of any mHealth application 3 months after the intervention; this was not limited to the 6 applications recommended in the intervention. The secondary outcomes were the frequency of mHealth application usage, improvements in dietary and exercise habits, and weight loss after 3 months and changes in anthropometric measurements and clinical laboratory test results after 1 year. Adverse events were reported to the complaint officer at each institution and promptly transmitted to the study office.

### 2.7. Sample size

Initially, we assumed that the proportion of mHealth application users in the general population would be 5%, which would increase to 10% following the intervention. Based on these assumptions, the required sample size was calculated using the Fisher exact test with a 2-sided significance level of 5% and a statistical power of 80%, resulting in 466 participants per group (total: 932 participants). After deducing expected potential dropouts, we set the target to 500 participants per group or 1000 participants in total. However, achieving the initially planned number of participants was difficult due to behavioral restrictions associated with the coronavirus disease 2019 (COVID-19) pandemic. Therefore, the feasibility of the study was re-examined before analyzing the outcome data by reviewing the existing literature and considering the expected effect size under pandemic conditions. Based on a previous study,^18^ the proportion of mHealth application users in the intervention group could increase to approximately 20%, particularly given the heightened interest in digital health tools during the stay-at-home period. Based on this reassessment, we judged that the number of participants ultimately recruited (*n* = 156) would still provide meaningful insights into the potential effectiveness of the intervention.

### 2.8. Statistical analysis

Continuous variables were expressed as medians (interquartile range [IQR]) or means (95% CI), and nominal variables were expressed as frequencies (%), unless stated otherwise. Intergroup differences in baseline values of continuous variables were assessed using the Mann-Whitney *U* test, and those for nominal variables were assessed using the Fisher exact test. The primary outcome (the usage of mHealth applications after 3 months) was compared between the groups using the Fisher exact test. The secondary outcomes (changes and change rates) were compared between the groups using an unpaired 2-sample *t* test, and within-group comparisons were performed using a paired *t* test. Between-group comparisons of the frequency of mHealth application use were performed using the Mann-Whitney *U* test. Within-group comparisons of nominal variables were performed using the McNemar test, and between-group comparisons were performed using the Fisher exact test. No multivariable analyses were performed due to the limited sample size.

Additionally, since a baseline imbalance in age was observed between the groups, we conducted a post hoc sensitivity analysis using logistic regression, with age as a covariate and mHealth application usage at 3 months as the dependent variable. For 2 applications—Aruku& and CALO mama Plus—where usage logs were available, we assessed the concordance between self-reported application usage and actual usage logs using Cohen kappa coefficient.

All analyses were performed according to the intention-to-treat principle based on the full analysis set, which included all participants who were enrolled, randomized, received the initial interview, and had valid baseline data. All analyses were performed using SPSS Statistics version 28 (IBM Corp., Armonk, NY, USA), with *P* < .05 indicating statistical significance.

## 3. Results

**Figure 1:**
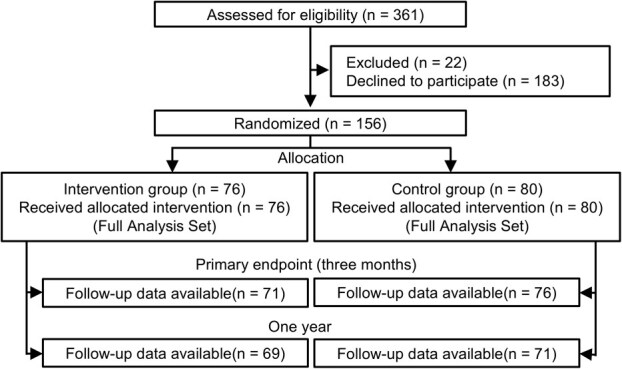
Selection of study participants.

Figure 1 presents the selection of study participants. Overall, 156 eligible participants were enrolled in the study, and 76 and 80 participants were allocated to the intervention and control groups, respectively. Five and 4 participants in the intervention and control groups were lost to follow-up. Therefore, 71 (93.4%) and 76 (95.0%) participants in the intervention and control groups, respectively, completed the trial and were included in the analyses. The participants’ baseline characteristics are presented in Table 2. No significant differences were seen in the participants’ demographic information (except for age), medical conditions, or mHealth application use between the 2 groups.

**Table 2:** Baseline characteristics of the participants.

	**Total (*n* = 156)**	**Intervention group (*n* = 76)**	**Control group (*n* = 80)**	***P* value**
Age, median (IQR), y	51.0 (46.0, 56.0)	48.5 (45.0, 56.0)	53.0 (48.0, 56.8)	.031
Sex, female, *n* (%)	39 (25.0)	17 (22.4)	22 (27.5)	.579
Body weight, median (IQR), kg	74.6 (69.3, 79.0)	74.6 (68.9, 78.1)	74.5 (69.6, 80.9)	.486
Body mass index, median (IQR), kg/m^2^	26.1 (25.0, 28.2)	26.0 (24.6, 27.6)	26.4 (25.1, 28.6)	.121
Waist circumference, median (IQR), cm	90.0 (86.5, 95.0)	89.7 (86.2, 94.2)	90.0 (86.9, 95.1)	.513
Blood pressure, median (IQR), mmHg				
Systolic blood pressure	124.0 (117.0, 135.0)	126.0 (118.0, 137.8)	123.0 (116.3, 134.0)	.299
Diastolic blood pressure	80.0 (73.3, 88.0)	81.0 (76.0, 89.0)	77.0 (72.3, 86.8)	.088
Fasting plasma glucose, median (IQR), mg/dL	97.0 (91.0, 103.0)	96.0 (89.3, 105.0)	98.0 (94.0, 103.0)	.242
HbA1c, median (IQR), %	5.6 (5.4, 5.7)	5.5 (5.4, 5.8)	5.6 (5.4, 5.7)	.888
LDL cholesterol, median (IQR), mmol/L	141.5 (120.0, 159.0)	142.0 (120.3, 159.0)	140.0 (117.8, 158.8)	.742
HDL cholesterol, median (IQR), mmol/L	55.9 (48.0, 64.0)	56.0 (49.0, 63.8)	55.4 (47.4, 64.0)	.847
Triglyceride, median (IQR), mmol/L	111.5 (81.0, 145.0)	114.5 (81.0, 145.0)	110.5 (81.75, 145)	.689
mHealth application usage, *n* (%)				
Any	66 (42.3)	33 (43.4)	33 (41.2)	.871
Pedometer	57 (36.5)	27 (35.5)	30 (37.5)	.868
Weight management	21 (13.5)	9 (11.8)	12 (15.0)	.643
Exercise	9 (5.8)	3 (5.8)	6 (7.5)	.496
Diet	7 (4.5)	3 (3.9)	4 (5.0)	>.999
Sleep	6 (3.8)	3 (3.9)	3 (3.8)	>.999
Blood pressure management	6 (3.8)	3 (3.9)	3 (3.8)	>.999
Blood glucose management	1 (0.6)	1 (1.3)	0 (0.0)	.487
Others	6 (3.8)	4 (5.3)	2 (2.5)	.434
Frequency of mHealth application use, median (IQR), d/wk	0.0 (0.0, 6.5)	0.0 (0.0, 7.0)	0.0 (0.0, 4.8)	.606

Abbreviations: HbA1c, glycated hemoglobin; HDL, high-density lipoprotein; IQR, interquartile range; LDL, low-density lipoprotein.

For the intervention group, the health care professionals recommended the following applications using the Study Guide algorithm: BeatFit (14 participants), Aruku& (27 participants), CALO mama Plus (23 participants), Genshu Nikki (9 participants), Minchalle (2 participants), and Bodygram (18 participants). Two or more applications were presented to some participants.

Table 3 presents the study outcomes. After 3 months, 52 participants in the intervention group (68.4%) and 32 participants in the control group (40.0%) were using mHealth applications, indicating a significant difference (*P* < .001). The weekly frequency of usage was also significantly greater for the intervention group (4.0 d/wk; IQR, 0.0, 7.0 d/wk) than the control group (0.0 d/wk; IQR, 0.0, 0.0 d/wk). Regarding applications where usage logs were available, a reasonable level of consistency was observed between participants’ self-reported usage and the system-recorded usage logs (Supplementary Material 3). No significant differences were seen in the proportion of participants reporting improvements in dietary or exercise habits. In the intervention group, body weight decreased compared with the baseline, whereas the control group did not exhibit a significant reduction. However, no significant difference was observed between the 2 groups (intervention group: −0.85 kg [95% CI, −1.66 to −0.04 kg]; control group: −0.19 kg [95% CI, −1.00 to 0.62 kg]).

**Table 3:** Changes in the proportion and frequency of application usage, behavior, and body weight after 3 months.

**Outcomes**	**Intervention group *n* = 76**		**Control group *n* = 80**		**Difference between groups (95% CI)**	***P* value**
		***P* value**		***P* value**		
mHealth application^a^						
Usage, *n* (%)	52 (68.4)	—	32 (40.0)	—	3.25 (1.68 to 6.28)^d^	<.001
Change in usage, *n* (%)	19 (25.0)	.002	−1 (−1.3)	.763	—	—
Frequency of use, median (IQR), d/wk	4.0 (0.0, 7.0)	—	0.0 (0.0, 7.0)	—	0.0 (0.0 to 1.0)^e^	.005
Behavior^b^						
Improvement of dietary habits, *n* (%)	35 (46.1)	—	37 (46.3)	—	0.99 (0.53 to 1.86)^d^	1.000
Improvement of exercise habits, *n* (%)	28 (36.8)	—	31 (38.8)	—	0.92 (0.48 to 1.76)^d^	.869
Anthropometric measurements^c^						
Change in body weight, mean (95% CI), kg	−0.85 (−1.66 to −0.04)	.040	−0.19 (−1.00 to 0.62)	.646	−0.66 (−1.79 to 0.47)	.252

Abbreviation: IQR, interquartile range.

a mHealth application usage data at 3 months were available for 71 participants in the intervention group and 76 participants in the control group.

b Questionnaires about behavioral change were completed by participants in the intervention and control groups (69 participants each).

c Anthropometric measurements at 3 months were available for participants in the intervention and control groups (70 participants each).

d Odds ratio.

e Hodges-Lehmann estimator.

Table 4 presents the changes in the anthropometric measurements and laboratory test results after 1 year. The members of both groups lost weight over the course of the year (intervention group: −0.88 kg [95% CI, −1.75 to −0.01 kg]; control group: −0.67 kg [95% CI, −1.29 to −0.07 kg]). Waist circumference changed by −0.9 cm (95% CI, −1.8 to −0.1 cm) and −0.4 cm (95% CI −1.1 to 0.2 cm) for the intervention and control groups, respectively. However, no significant differences were observed in weight loss or waist circumference reduction between the groups. The blood pressure, fasting plasma glucose, HbA1c levels, high-density lipoprotein cholesterol, and low-density lipoprotein cholesterol showed minimal changes in both groups. However, the triglyceride levels decreased in the intervention group, whereas they increased in the control group (intervention group: −10.9 mg/dL [95% CI, −22.8 to 0.9 mg/dL]; control group: 14.3 mg/dL [95% CI, −1.6 to 30.2 mg/dL]; intergroup *P* = .013).

**Table 4:** Changes in anthropometric measurements and laboratory data after 1 year.^a^

	**Intervention group**		**Control group**		**Difference between groups (95% CI)**	***P* value**
	**Mean (95% CI)**	***P* value**	**Mean (95% CI)**	***P* value**		
Anthropometric measurements						
Body weight, kg	−0.88 (−1.75 to −0.01)	.047	−0.67 (−1.29 to −0.07)	.029	−0.20 (−1.25 to 0.84)	.701
Waist circumference, cm	−0.9 (−1.8 to −0.1)	.031	−0.4 (−1.1 to 0.2)	.191	−0.5 (−1.6 to 0.6)	.358
Blood pressure, mmHg						
Systolic blood pressure	0.3 (−2.6 to 3.2)	.841	0.9 (−1.8 to 3.6)	.506	−0.6 (−4.6 to 3.3)	.753
Diastolic blood pressure	0.0 (−1.8 to 1.9)	.976	0.1 (−2.0 to 2.2)	.947	−0.0 (−2.8 to 2.8)	.977
Laboratory data						
Fasting plasma glucose, mg/dL	−1.7 (−3.5 to 0.1)	.058	−0.2 (−2.2 to 1.8)	.819	−1.5 (−4.1 to 1.1)	.273
HbA1c, %	0.03 (−0.01 to 0.07)	.156	0.07 (0.01 to 0.11)	.006	−0.03 (−0.10 to 0.03)	.252
LDL cholesterol, mg/dL	−2.3 (−7.8 to 3.2)	.406	−5.0 (−11.3 to 1.3)	.116	2.7 (−5.6 to 11.0)	.522
HDL cholesterol, mg/dL	1.2 (−0.4 to 2.8)	.149	1.3 (−0.4 to 3.0)	.135	−0.1 (−2.4 to 2.2)	.923
Triglycerides, mg/dL	−10.9 (−22.8 to 0.9)	.071	14.3 (−1.6 to 30.2)	.076	−25.2 (−45.0 to −5.5)	.013

Abbreviations: HbA1c, glycated hemoglobin; HDL, high-density lipoprotein; LDL, low-density lipoprotein.

a Anthropometric measurements and laboratory data at 1 year were available for 69 participants in the intervention group and 71 participants in the control group.

Considering the baseline age imbalance between the groups, a sensitivity analysis using logistic regression was performed with age as the independent variable and mHealth application usage at 3 months as the dependent variable. Even after adjusting for age, being assigned to the intervention group remained a significant predictor of mHealth application usage (Supplementary Material 4).

No significant adverse events were observed during the intervention period.

## 4. Discussion

This study demonstrated that advising participants to use commercially available mHealth applications in a health guidance setting significantly increased the number of users of mHealth applications and the frequency of mHealth application usage after 3 months, along with a significant reduction in triglyceride levels after 1 year.

In this study, the intervention group showed a relatively high usage rate of 68.4% after 3 months, exceeding the rate reported in previous studies.^18^^,^^19^ This may be due to the already high baseline usage rate of 42.3%, which may reflect heightened interest in digital health tools during the COVID-19 pandemic, and the inclusion of a broad range of commercially available mHealth applications in the analysis. Additionally, several features of the intervention may have contributed to the further increase observed in the intervention group. Individualized recommendations and setup guidance were provided during a health guidance session, which may have facilitated smooth adoption. Furthermore, enabling participants to select from multiple applications suitable for their specific behavioral goals, digital literacy, and preferences may have supported higher engagement and retention. Although the use of mHealth applications is associated with better lifestyle habits,^20-23^ a major challenge to studying their benefits is their low retention rate.^18^^,^^19^^,^^24^^,^^25^ In this study, sustained application usage may have been supported by tailored advice and support from health care professionals and the availability of personalized information, which are known to enhance the effectiveness of web-based health interventions.^26^

This study targeted individuals with a history of participating in the SHG program. Our previous cohort study showed that standard health guidance may be less effective in this group.^6^ These individuals may have already been exposed to conventional advice, and additional or alternative approaches may be required for further improvement. In such cases, particularly in settings such as Motivational Health Guidance, where human and financial resources are limited, incorporating low-cost and scalable interventions is crucial. The use of commercially available mHealth applications is highly effective with respect to reducing costs and human resources.^27^ Although initial support for introducing an application requires some effort, the self-monitoring and automatic feedback (often artificial intelligence–driven) functions of mHealth applications can ensure repeated usage without the ongoing assistance of health care professionals. Furthermore, the use of commercially available applications rather than developing and testing an original application prevented unnecessary application development costs. Well-designed commercially available mHealth applications can prevent user dropout through gamification and feedback from the applications.^28^ In health guidance settings, such as Motivational Health Guidance and counseling sessions with occupational health nurses or industrial physicians, where the sessions often amount to only a single interview, incorporating such application-based interventions can be a practical option. mHealth applications are easy to use, do not require much time, and are not time-restrictive, making them attractive options for the busy middle-aged working population.

In this trial, a guide for health care professionals was created and used to facilitate smooth presentation and support.^17^ This guide was organized to assist health care professionals, even those unfamiliar with introducing mHealth applications, in selecting appropriate applications based on the participants’ behavioral goals and providing effective guidance for lifestyle improvement using these applications. This support can help reduce the burden in health guidance settings. Moving forward, it would be beneficial to develop and widely disseminate training materials for health care professionals that they can use to support the effective introduction and use of various mHealth applications in health guidance settings.

Dietary health applications provide guidance on the appropriate intake of certain nutrients, including carbohydrates and fats, whereas exercise and alcohol reduction applications promote aerobic activity and reduced alcohol consumption, respectively. Notably, however, in this study, no differences were observed after 3 months between the intervention and control groups with respect to dietary and exercise habits. However, a significant decrease in triglyceride levels was observed only in the intervention group after 1 year. This may be because certain improvements in lifestyle habits observed at 3 months might have persisted longer in the intervention group. Alternatively, delayed or qualitative behavioral changes might have occurred in the intervention group, ultimately contributing to reduced triglyceride levels. Conversely, the increase in triglyceride levels observed in the control group may reflect the natural progression of metabolic disorders that were prevented in the intervention group. Nevertheless, as triglyceride was one of several secondary endpoints assessed in the study, the possibility of a statistical error due to multiple comparisons should be considered. A detailed, long-term evaluation of application use and lifestyle improvements is needed to confirm this.

This study has several limitations. First, as mHealth application usage and behavioral changes were based on self-reported data, social desirability bias and the Hawthorne effect may have influenced the results. Therefore, participants were not informed that application usage was the primary outcome. Additionally, usage logs for some applications were available and generally consistent with self-reported application usage, indicating the reliability of the data. However, future studies should incorporate objective measures, such as comprehensive usage logs and activity trackers, to improve accuracy. Second, the COVID-19 pandemic may have affected the participants’ daily behavior and the study results. Third, mHealth application usage was assessed at baseline and 3 months after the intervention. Therefore, short-term usage patterns during the 3-month period were not captured, and the number of new users or their continuation status could not be determined. Additionally, the effects of sustained usage on outcomes beyond 1 year remain unclear. Therefore, further studies with more frequent and longer-term follow-ups are needed to address these limitations.

## 5. Conclusion

This study found that providing advice regarding the use of mHealth applications that is tailored toward users’ individual behavioral goals in a health guidance setting significantly increased the number of users and the frequency of application use and improved triglyceride levels. These findings indicate that the use of commercially available mHealth applications can enhance the effectiveness of health guidance programs, especially in environments with limited human and financial resources.

## Supplementary Material

Web_Material_uiaf036

## Data Availability

The datasets used and/or analyzed during the current study are available from the corresponding author on reasonable request.
