# Supplementary material for: Effectiveness of recommendations in promoting the use of mobile health applications in health guidance: a randomized controlled trial
Source: J Occup Health. 2025 Jul 17;67(1):uiaf036. doi: 10.1093/joccuh/uiaf036 (PMC12321321; doi:10.1093/joccuh/uiaf036)
Supplement: Web_Material_uiaf036 [file web_material_uiaf036.pdf]

Table of contents

Supplementary Material 1. Features of each mHealth application

Supplementary Material 2. Algorithm for determining the recommended mHealth applications:Guide for health instructors (excerpts)

Supplementary Material 3. Agreement between application usage logs and self-reported application usage among Aruku& and CALO mama Plus users

Supplementary Material 4. Sensitivity analysis using logistic regression of mHealth application usage after 3 months, with adjustment for group allocation and age

Supplementary Material 1. Features of each mHealth Application

	BeatFit<br>Aristol Inc.	aruku&<br>ONE COMPATH CO., LTD.	CALO mama plus<br>Wellmira Inc.
**Characteristics**	An application that allows users to enjoy exercising anytime and anywhere with voice guidance from professional trainers and popular background music. With over 650 classes across 11 genres, including strength training, stretching, yoga, meditation, and sleep, even novice users can operate it with confidence.	A walking application that ramifies the experience. Users can earn points on the basis of their steps, collect characters, and redeem points for raffles offering various prizes, including convenience store coupons or local specialty products, encouraging continued walking.	This application enables users to take photos of their meals and instantly receive artificial intelligence (AI)-generated analyses of calories and nutrients, along with individualized dietary advice. Furthermore, it provides real-time recommendations on exercise, sleep, and mood logs. Daily summaries are provided to encourage behavioral changes.
**Guidance for Successful Use**	Suggest starting with the recommended menus that appear on the HOME screen and trying out the beginner classes first.	Encourage users to engage with the application in an engaging and gamified approach.	To increase the likelihood of successful weight loss, aim for at least four inputs per week and a health score of ≥60 points.
**Goal and Course Settings**		◎ (Setting target step counts)	◎ (Various course programs)
**Personalization**	◎ (Optimal program suggestions by AI)	◎ (Weekly update of target step count)	◎ (Preregistration of favorite menus, search history, and individual advice from AI dietitians based on input content)
**Push Notifications**	◎	◎	◎
**Gamification**	◎ (Coin rewards and challenge programs)	◎ (Character acquisition and prize incentives)	◎ (Point incentives)
**Feedback**	◎ (Class logs and cheering voice)	◎ (Step count trend graph)	◎ (Comments after each meal, daily scores, and chatbot)
**Peer Support**			
**Cost**	JPY 1,480 per month	Free of charge	Free of charge for users

Genshu Nikki<br>Otsuka Pharmaceutical Co.,Ltd.	Minchalle<br>A10 Lab Inc.	Bodygram<br>Bodygram Inc.
This application enables you to manage your average daily alcohol consumption and track changes in your daily intake using graphs. Based on the criteria for heavy drinking (males, 60 g/day; females, 40 g/day), this application clearly displays your intake on a calendar with illustrations. The amount of alcohol can be easily calculated by selecting the type and quantity from the illustrations.	This habit-forming application is recommended for those who tend to give up easily. Users can join a team of five anonymous members working toward the same goal and share photos and comments about their daily activities in a group chat. Users can work toward forming habits by encouraging one another. Even those who struggle to independently maintain habits can enjoy building them with the support of others.	This AI-powered body measurement application can assess 24 body areas within seconds using two photos as follows: one from the front and one from the side. Along with measuring waist circumference to assess metabolic syndrome, this application provides full-body measurements, enabling you to understand the effects of your daily diet and exercise not just by weight or body fat but also by your overall body shape.
Notify participants that they can input data at their discretion, whenever observations are made.	Highlighting the significance of habit formation.<br>Support the process from downloading the application to completing the tutorial, joining a team, and posting a test photo.	Inform participants to stand straight if someone else is taking the photo or place the camera on a vertical surface if taking a selfie. Moreover, inform the participants that they need not be naked for the photo and ensure that their head and feet are not cut off in the shot.
◎ (Set the participant’s target alcohol consumption)	◎ (Join a team that matches the participant’s goals)	
		◎(AI image analysis→body measurement)
◎	◎	
	◎ (Awarding coins and badges)	
◎ (Graphing of alcohol consumption trends)	◎ (Comments from team members, graphing of steps, and weight)	◎ (Graphing the trends in body measurements)
	◎ (Encouragement from team members toward the same goal).	
Free of charge	The application offers both a free plan and a paid plan (JPY 500/month)	Free of charge

Supplementary Material 2. Algorithm for determining the recommended mHealth applications

Guide for health instructors (excerpts)

Diet-Related Behavioral Goals

Examples:
“Eat until 80% full,” “Eat vegetables first.”
“Reduce snacks,” “Switch to healthier drinks.”

Points for Application Introduction Based on Diet-Related Behavioral Goals

How about keeping a food record and “visualizing” it?

Have you ever had these experiences while attempting to implement your behavioral goals?

- “Eat until 80% full” ⇒ I think I am eating less, but my weight is not changing...
- “Eat vegetables first” ⇒ I am eating vegetables first, but my weight is not changing...
- “Reduce snacks” ⇒ I am not eating sweets, but my weight is not changing...
- “Switch to healthier drinks” ⇒ I switched to drinks that seem healthier, but my weight is not changing...

If you are attempting hard to reduce your food intake but your weight is not decreasing, you may be performing it wrong!

Using CALO mama plus, you can understand your daily “calorie balance” to observe if your intake is not exceeding your consumption.

Tips for Introducing Applications

Confidence in Behavior Change

High: “This application has a high success rate for those who score an average of ≥60 points on their health score and record four or more times per week.”

Suggested Application: CALO mama plus

Low: “Attempt recording only on weekdays when daily recording is difficult.”
Suggested Application: Calomama Plus

“How about recording just your alcohol consumption?”
Suggested Application: Genshu Nikki

Target Degree of Weight Loss

High: “Recording your diet is highly effective for weight loss; therefore, please consider recording your meals using an application.”
Suggested Application: CALO mama plus

Low: “Adding exercise to your diet to build strength (muscle) and a great physique is also recommended.”
Suggested Applications: BeatFit, aruku&, and Bodygram

Need for Support

High: “There is an application wherein everyone can support each other to achieve their goals together.”
Suggested Application: Minchalle

Low: “There are also other health applications available.”
Suggested Material: Application guide leaflets

Use of Smartphone (Applications)

Game: “You can collect stamps and apply for fantastic products.”
Suggested Application: CALO mama plus

Social Network Service: “There is an application that allows you to encourage others with the same goals.”
Suggested Application: Minchalle

Hardly using it: Support application downloads focused on how to use them.
For those who are resistant to the use of applications:
Recommend checking their “step count” using the basic functions of their smartphones.
→ Android “Google Fit”; iOS “Health (Apple)”

Physical Activity and Exercise-Related Behavioral Goals

Examples:

"Increase step count," "Perform strength training," and "Want to flatten my stomach."

Points for Application Introduction Based on Physical Activity and Exercise Behavioral Goals

Point 1

First, we must understand the current situation. Determine whether you walk >60 min /day, have an established exercise habit, or possess sufficient physical strength. If the answer to any of these queries is "No," then start with "+10 (Plus Ten)." Move your body for 10 min/day by just performing anything.

Point 2

If you are already somewhat active, incorporate an additional +10. Aim to move your body energetically for 60 min/day if possible.

Point 3

If you have already achieved the habit of exercising or walking 60 min/day, share +10 with as many family members and friends as possible. Be thorough in managing your diet.

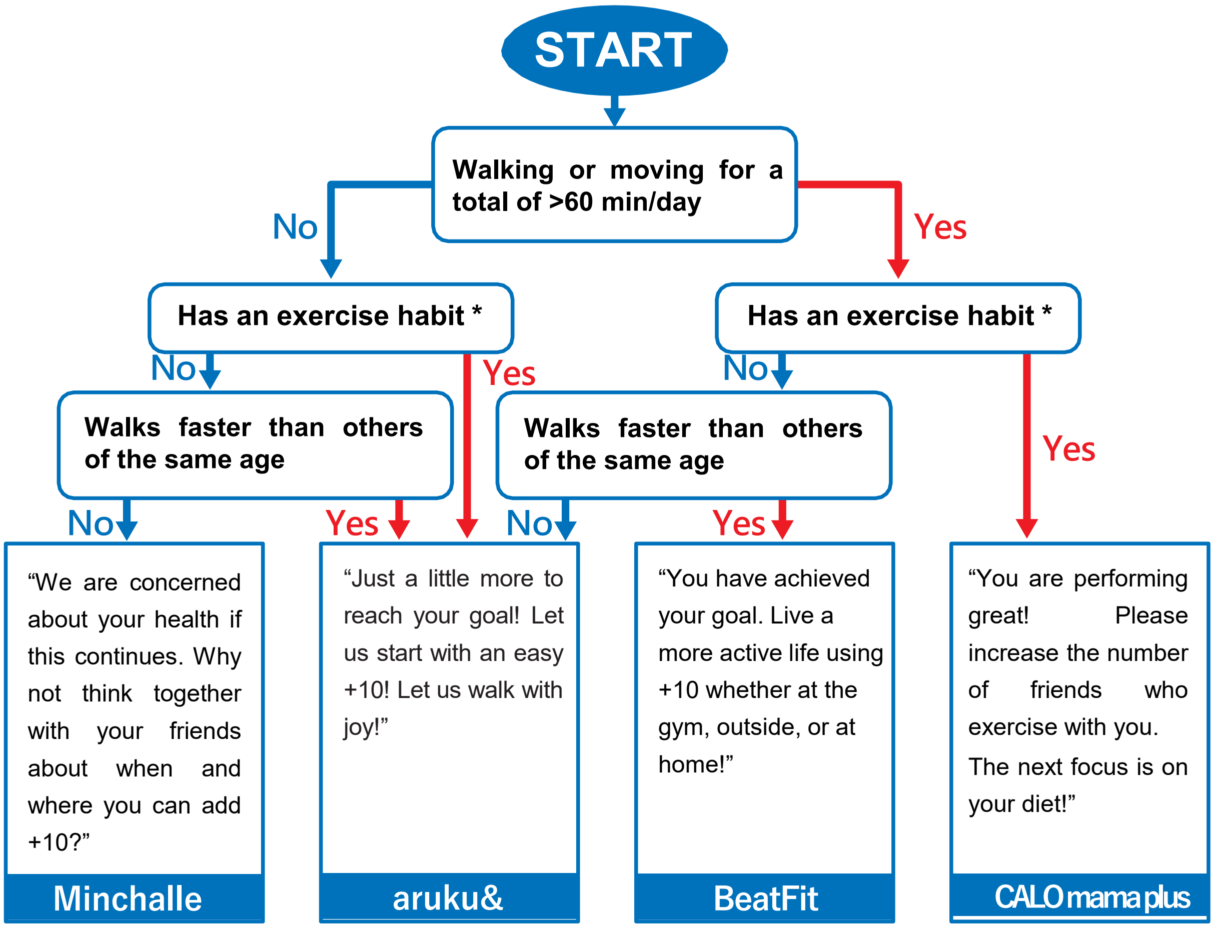

*Has been performing light sweat-inducing exercises for >30 min at least 2 days/week and has been continuing this for >1 year.

Alcohol Consumption-Related Behavioral Goals

Examples:

“Reduce alcohol consumption”

“Have alcohol-free days”

Points for Application Introduction Based on Alcohol Consumption Behavioral Goals

Point 1: To abstain or to reduce drinking? That is the question!

Abstinence is recommended for individuals with alcohol dependence, whereas reduced drinking is advised for others . Among those who drink every day without alcohol-free days, hidden alcohol dependence may exist. The AUDIT test is employed for screening problem drinkers.

Point 2: Communicate the appropriate moderate drinking amount

Appropriate moderate drinking is approximately 20 g of pure alcohol per day on average. Drinking that increases the risk of lifestyle-related diseases is 40 and 20 g/day for males and females, respectively , and excessive drinking is ≥60 g/day.

Point 3: Encourage future-oriented questions and record-keeping

Ask future-oriented questions, such as “If you think about your future health, will you establish alcohol-free days or will you reduce the amount you drink at one time?,” and encourage keeping records using an application.

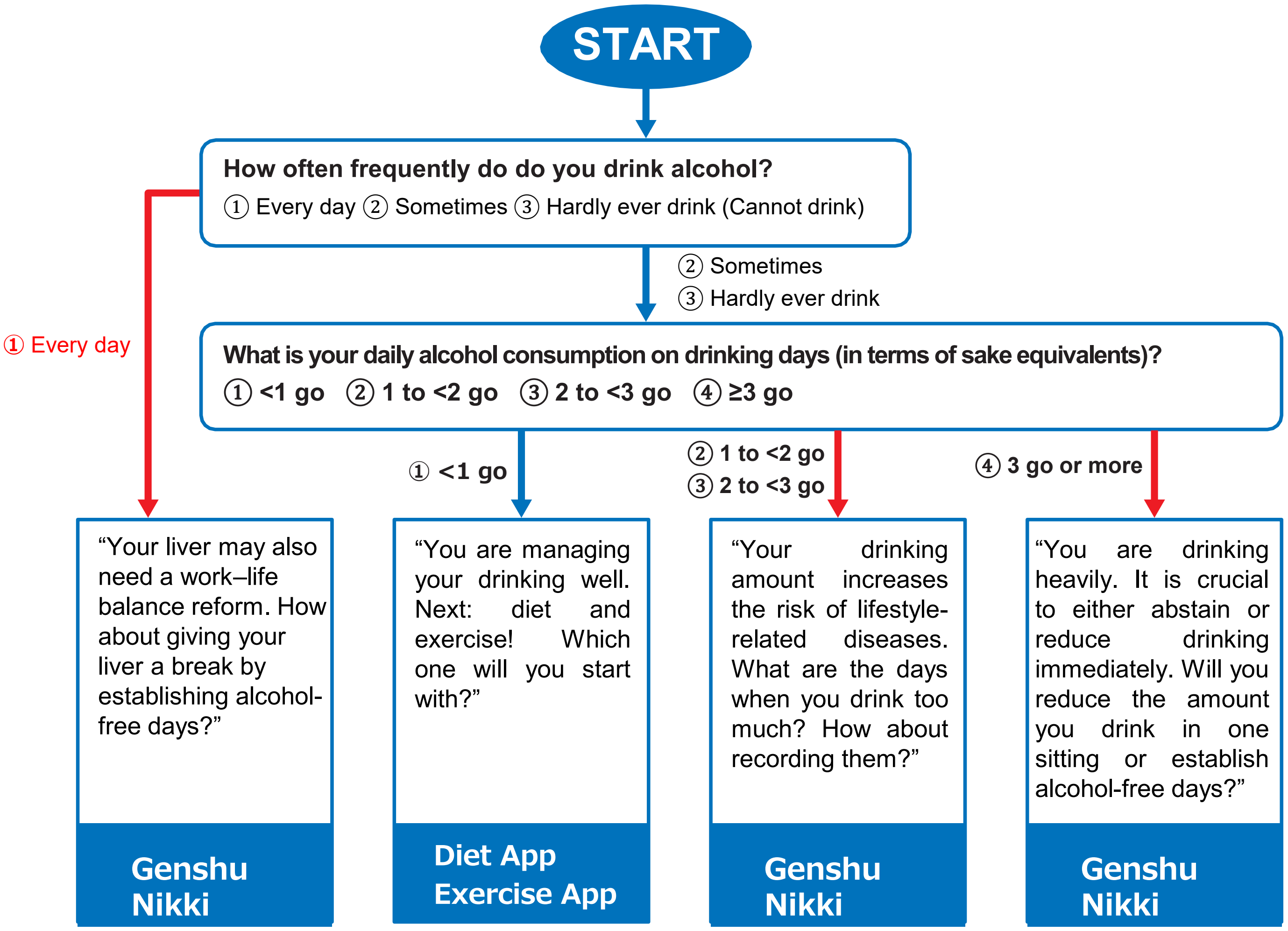

(Note: “go” is a traditional Japanese unit of volume commonly used for measuring sake. One go is approximately 180 mL or 6.1 fl oz).

Supplementary Material 3. Agreement between application usage logs and self-reported application usage among Aruku& and CALO mama Plus users

mHealth application	Self-report–Log agreement rate	Cohen's kappa (95%CI)
Aruku&	88.0% (22/25)	0.59 (0.18, 1.00)
CALO mama Plus	81.2% (13/16)	0.45 (−0.06, 0.97)

Agreement between the presence of application usage logs at 3 months and self-reported app usage at 3 months was evaluated. Analysis was conducted among Aruku& users (n = 25) and CALO mama Plus users (n = 16) who had available app usage logs and completed the 3-month questionnaire. CI, confidence intervals.

Supplementary Material 4. Sensitivity analysis using logistic regression of mHealth application usage after 3 months, with adjustment for group allocation and age

	OR (95%CI)	*p*-value
Group		
Control group	Reference	
Intervention group	3.09 (1.59, 6.01)	<0.001
Age	0.97 (0.92, 1.03)	0.309

Logistic regression analysis using health-related app usage at 3 months as the dependent variable for sensitivity analysis. Group allocation and age were included as explanatory variables. OR, odds ratio; CI, confidence intervals.
